# A Deep Intelligent Attack Detection Framework for Fog-Based IoT Systems

**DOI:** 10.1155/2022/6967938

**Published:** 2022-12-22

**Authors:** Surya Pavan Kumar Gudla, Sourav Kumar Bhoi, Soumya Ranjan Nayak, Krishna Kant Singh, Amit Verma, Ivan Izonin

**Affiliations:** ^1^Department of Computer Science and Engineering, NCR-PMEC Berhampur, Faculty of Engineering, BPUT, Rourkela 769015, Odisha, India; ^2^Department of Computer Science and Engineering, Parala Maharaja Engineering College (Govt), Berhampur 761003, Odisha, India; ^3^School of Computer Engineering, KIIT Deemed to be University, Bhubaneswar 751024, Odisha, India; ^4^Department of CSE, ASET, Amity University, Noida 201301, India; ^5^Department of Computer Science & Engineering and University Center for Research and Development, Chandigardh University, Mohali 140413, Punjab, India; ^6^Department of Artificial Intelligence, Lviv Polytechnic National University, Lviv 79013, Ukraine

## Abstract

Fog computing provides a multitude of end-based IoT system services. End IoT devices exchange information with fog nodes and the cloud to handle client undertakings. During the process of data collection between the layer of fog and the cloud, there are more chances of crucial attacks or assaults like DDoS and many more security attacks being compromised by IoT end devices. These network (NW) threats must be spotted early. Deep learning (DL) assumes an unmistakable part in foreseeing the end client behavior by extricating highlights and grouping the foe in the network. Yet, because of IoT devices' compelled nature in calculation and storage spaces, DL cannot be managed on those. Here, a framework for fog-based attack detection is proffered, and different attacks are prognosticated utilizing long short-term memory (LSTM). The end IoT gadget behaviour can be prognosticated by installing a trained LSTMDL model at the fog node computation module. The simulations are performed using Python by comparing LSTMDL model with deep neural multilayer perceptron (DNMLP), bidirectional LSTM (Bi-LSTM), gated recurrent units (GRU), hybrid ensemble model (HEM), and hybrid deep learning model (CNN + LSTM) comprising convolutional neural network (CNN) and LSTM on DDoS-SDN (Mendeley Dataset), NSLKDD, UNSW-NB15, and IoTID20 datasets. To evaluate the performance of the binary classifier, metrics like accuracy, precision, recall, f1-score, and ROC-AUC curves are considered on these datasets. The LSTMDL model shows outperforming nature in binary classification with 99.70%, 99.12%, 94.11%, and 99.88% performance accuracies on experimentation with respective datasets. The network simulation further shows how different DL models present fog layer communication behaviour detection time (CBDT). DNMLP detects communication behaviour (CB) faster than other models, but LSTMDL predicts assaults better.

## 1. Introduction

IoT gadgets like IoVT, IoMT, smart grids, and smart electrical appliances, inter alia, are exceedingly raising in current technologies, leading to so many attacks on those devices with their prominence in resource sharing. Physical devices like sensors and actuators give on-demand administration over the cloud, but its centralization is hazardous. All with this, on providing services by cloud to IoT faces high challenges in data abeyance, data security, data obtrusion, and data shielding [1–8].

An abstraction layer called Fog is used to offer services close to the network's edge and solve cloud-based IoT challenges. Fog, a distributed decentralized model, has evolved that lies between the cloud and client devices [2], in providing services with less latency and less bandwidth utilization in the network (NW). Howbeit, the fog nodes have a low level of data privacy and are vulnerable to assaults such as probe, DDoS, man-in-the-middle, port scan attacks, and many others [9]. As a result, the fog layer needs an attack detection system. Noncellular network protocols including LoRa, COAP, LoRaWAN, and MQTT, are required to enable communication between the smart devices. These protocols help end users by having low latency and low bandwidth utilization [5, 10]. Data collected from end devices gets exchanged and set aside for the devices using fog communication protocols for further speedy retrieval of data. During the exchange of data, the fog layer/fog-node is more susceptible to attacks. Hence, we require a security system in the fog layer to define attack detection. This work uses LSTMDL model to prognosticate fog layer attacks.

Likewise with immense expansion in use of web and huge measure of information move, has caused a more noteworthy number of peculiarities. In equal measure, the reason for attacks is additionally expanding reliably. Numerous associations are consistently working on network attack discovery to offer secure types of aid to end users. Because of high utilization of cloud administrations and IoT over fog layer prompts more expanded hazard of information infringement. Here in such manner compelled to give or configure more secured system by DL algorithms which can distinguish the attacks powerfully.

With truly expanding of web, the general public is moving towards present day advancements to foresee, recognize or order and investigation network conduct using ML and DL approaches are broadly utilized. Henceforth attack detection is turning out to be latest pattern and examination scope for cyber threats.

Due to geo-distribution and location awareness fog layer became exacting in its nature. At first to distinguish attacks ML strategies are profoundly utilized yet inadmissible for enormous magnitude of information. To beat the limit of ML, DL is utilized in distinguishing assaults in the fog layer as it has numerous layers in handling with a high detection rate. On detection of an attacker, the fog node sends the behaviour update of the node to the cloud as malicious and nonmalicious and multilabel classification [1, 4, 9, 11–15].

With a premier detection rate, DL has been used to categorize numerous attacks, producing binary classifications of typical and aberrant behaviour as well as multilabel classifications that are sent to the cloud for node behaviour updates [1, 4, 9, 11, 12]. Because of the resource restraint nature of IoT, it is preposterous to expect to execute complex DL calculations. Along these lines, DL is reasonable to carry out on fog node/fog layer with high precision. In light of the enormous amount of data, DL is superior to ML calculations. The LSTMDL model is used in this work to identify a security attack on an IoT application based on fog nodes.

This work's accomplishments are as follows:

1. For fog-based IoT systems, a deep intelligent attack detection framework is suggested in this paper. The framework uses the LSTMDL model to find NW security breaches.
2. The LSTMDL model is set up in a fog node's compute module to analyze the behaviour of end IoT devices. To choose the most accurate DL model at the fog layer, potentiality balancing is performed across HEM, Bi-LSTM, GRU, CNN + LSTM, DNMLP, and LSTM.
3. The experimentation is done by using Anaconda platform by considering SDN [16], NSLKDD [17], UNSW-NB-15 [18], and IoTID20 datasets [19] for different attacks.
4. From the upshots, it is found that the LSTMDL model showed finer accuracy than the other five models, and it is considered in this framework to prognosticate the attack. LSTMDL model shows outperforming nature in binary classification with 99.70%, 99.12%, 94.11%, and 99.88% performance accuracies on experimentation with respective datasets.
5. The network simulation is also performed to show the performance of different DL models for presenting the behaviour detection time at the fog layer. From this upshot, it is found that DNMLP shows smaller communication behaviour detection time (CBDT) than other models; however; the LSTMDL model performs better in predicting the attacks well. Along with, the CBDT gets reduced with the rise in the number of fog-nodes.

The following is a discussion of the remaining sections: The acknowledgements are presented in Section 2, the system design with network design and assault design are mentioned in Section 3, the problem statement is discussed in Section 4, proffered deep intelligent assault prognostication structure is described in Section 5, and the performance evaluation with simulation setup, results, and discussion is presented in Section 6. Lastly, Section 7 presents the conclusion.

## 2. Literature Review

Numerous studies on the subject are presented in this section, and they present the best DL techniques for an assault prognostication structure for fog-based IoT systems. Samy et al. [1] proposed a framework for attack detection on several cyber-attacks using DL technique resulted high detection rate in multi classification with 99.65% and 99.96% detection accuracy in binary classification, respectively. Lawal et al. [2] used signature- and anomaly-based methods designed framework with two modules for oddity detection. Obtained accuracy for binary and multi classification by module-2 with 99% and 97% for average recall, precision, and F1-score using XGBoost classifier. Module-2 showed six times lesser performance than module-1. Puthal et al. [3] proposed advanced research issues needed for fog architecture and raised chances of threats and discussed the overcoming of threats at each layer in a three-layered architecture. Sudqi Khater et al. [10] considered ADFA-LD and ADFA-WD datasets to address problems on latency, mobility support, and location awareness on cloud using MLP model of lightweight IDS, which resulted in 92%, 94%, and 95% F1-measure, accuracy, and recall on ADFA-WD dataset. Obtained F1-measure, recall, and accuracy with 74% using Raspberry Pi on ADFA-LD dataset. Bhushan's [20] DDoS attack defence framework is proposed on Kali Linux Machine using LOIC on TCP traffic, allowed only legal request for accessing on cloud by framing rules at fog layer using fog defender. Priyadarshini and Barik [21] designed new DDoS defence model using DLMs by obstructing malicious packets transferred to cloud in order to overcome DDoS attacks on ISCX 2012 IDS and CTU-B Botnet datasets. The experimentation resulted with accuracy of 98.88% accuracy with 10-fold cross validation scheme. Chaudhary et al. [11] made survey on domain of computing and inspect subsisting things related to privacy, security, confronts, limitations, and open directions of research. Douligeris and Mitrokotsa [9] discussed elaborately on segregation of DDoS attack system, advantages, disadvantages, and techniques of defence models. Potluri et al. [12] presented various algorithms like machine learning, deep learning, neural network, blockchain, software defined networks, and genetic algorithm in cloud environment for detection and prevention mechanism. Kalaivani and Chinnadurai [22] designed fog computing intrusion detection model using to predict attacks by CNN and LSTM on NSL-KDD dataset with 96.5% accuracy. To prevent from malicious users the model is deployed in fog layer. This model is used in predicting multiclass attack classifications. By taking into account a variety of criteria, Churcher et al. [23] performed a comparison of various machine learning techniques for binary and multilabel classification. Kilincer et al. [24] performed a comparative study using different ML algorithms on five different datasets, namely, CSE-CIC IDS-2018, UNSW-NB15, ISCX-2012, NSL-KDD, and CIDDS-001. On comparison, the decision tree classifier proved to be better than the remaining two classifiers, SVM and KNN. Many such related research works can also be found in [25–36].

The research gaps that are identified from the study on distinct attack detection frameworks are observed, for instance, (i) performance accuracy or exactness is evaluated on smaller datasets with fewer attributes which fall behind in better attack detection. Hence, we considered newer datasets DDoS-SDN [16] and IoTID20 [19] datasets with huge number of instances and attributes. (ii) Even with the increase in dataset size, most of the prognostications are made on conventional ML algorithms which do not yield better accuracy for attack detection and became cumbersome to decide best ML algorithm on selected datasets. (iii) From the observations on many datasets, we listed only fewer number of attacks and hence need to be considered dataset with more number of attacks which helps in better prognostication of attacks.

## 3. System Design

In this part, a framework is considered with both the NW and assault designs. The network model portrays about the organization part, the network arrangement, and the correspondence between the organization parts. Attack model portrays how the perpetrator attacks the organization. The notations used in this paper are shown in Table 1.

### 3.1. Network Design

**Figure 1:**
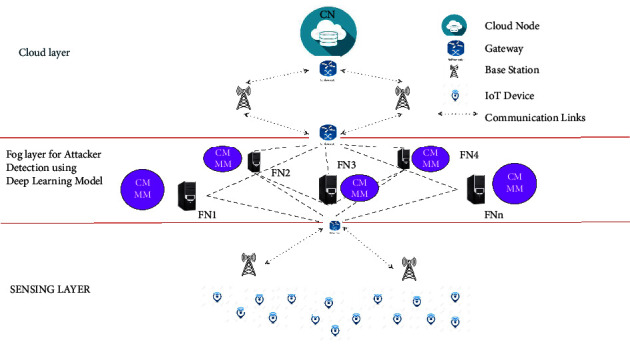
System architecture.

The network design is designed with a three-layered architecture containing cloud, fog, and IoT end devices in the top layer, amid layer, and bottom layer, respectively, [1–3, 23, 24] as depicted in Figure 1. The Cloud Node (CN), an upper layer that stores updated behaviours (attacker/normal) of the end devices as centralized data storage which is connected to amid layer through gateway (GW) and base station (BSS) using either wired or wireless communication.

The amid layer, called the secure fog layer, containing *k* number of fog nodes FN={FN_1_, FN_2_,…, FN_*k*_} which performs computations, localized communication, and data storage for the nearby IoT end device. They likewise record the way of behaving of the devices promptly. The FN for the most part comprises of a CMFN and MMFN. The CMFN of a FN is empowered where the CMFN is prepared with a DL model to play out an errand to anticipate the ways of behaving of the IoT devices which speaks with the FN in closeness. The FNs are likewise associated with one another through wired/remote interchanges for informational correspondence among them. The secure fog layer is associated with the upper cloud layer through GW and BSs. The correspondence happens utilizing wired/wireless interchanges. The secure fog layer is likewise associated with the lower layer utilizing GW and BSs, through which correspondence happens.

The lowest layer referred to as the sensing layer that mainly composed of IoT devices {iot_1_, iot_2_,…, iot_*l*_} which does enormous amount of end clients information or solicitations to fog or cloud for quick calculation and administration. For communication with the cloud or fog layer, the IoT devices use BSs and GT.

### 3.2. Assault Design

**Figure 2:**
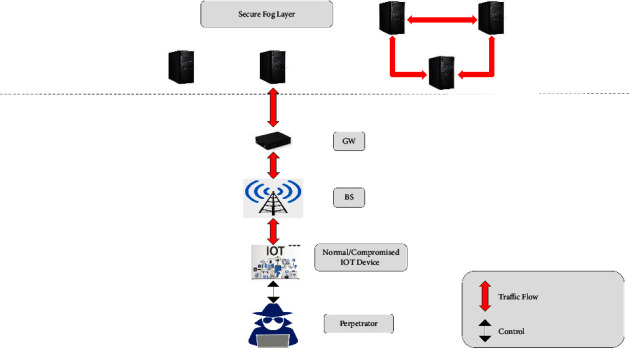
Assault design.

The peculiarity in the NW now and again causes diversion from the ordinary progression of traffic, which prompts an assault by the attacker *P*_*k*_. In a fog-based IoT environment, attackers may originate from IoT devices, protocols, applications, and software. Vulnerabilities can arise on various device parts such as web interface, memory, and firmware. Protocols in IoT end devices, by means of communication channels and related applications and software, are also prone to security issues and attacks [20, 21, 37].  Figure 2 shows a typical attack sequence model. By taking possession of the IoT devices that are connected to the closest fog node, the attacker launches various attacks on the fog nodes in the fog layer.

## 4. Problem Statement

The problem statement defined in the model with *l* number of IoT end devices as *I*={*i*_1_, *i*_2_,…, *i*_*l*_} communicates with *k* FNs with distinct communication behaviours denoted as CB={cb_1_, cb_2_,…, cb_*l*_}, where *k* ≤ *l* and each *cb*_*k*_ has set of communication instances (CI) at different time intervals (TI), represented as *CI*={ci_1_, ci_2_,…., ci_*m*_} and *TI*={*t*_1_, *t*_2_,…, *t*_*m*_} where *m* is the number of CI with distinct TI between IoT and FN. On communication, IoT with FN considers different attributes to obtain target label which is denoted as either normal (0) or attacker (1) from the dataset where the set of attributes is denoted as *A*={*a*_1_, *a*_2_,…, *a*_*p*_}. In this work, the main problem is to predict the behaviour of the IoT devices more accurately by training and testing on different standard datasets by implementing DNMLP, LSTM, Bi-LSTM, GRU, CNN + LSTM, and HEM DL models.

## 5. Proffered Deep Intelligent Assault Prognostication Framework

**Figure 3:**
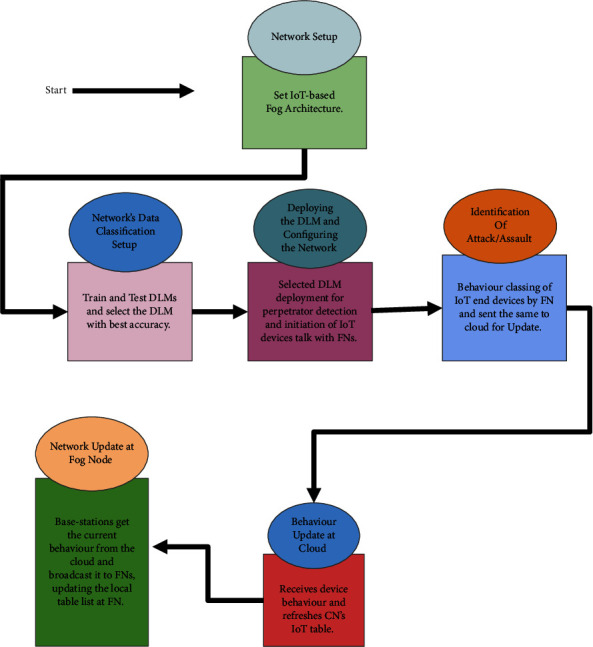
Flow of the proffered assault prognostication framework.

The proffered assault prognostication framework is presented here is designed to tackle the above issue. The framework principally comprises six stages: (1) network configuration/setup, (2) network's data classification setup, (3) deploying deep learning models and configuring the network, (4) identification of assault, (5) behaviour update at cloud, and (6) network update at FN. In Figure 3, the operational flow model of these six steps is shown.

### 5.1. Network Setup

The cloud CN is first set up as depicted in Figure 3 which offers various kinds of help to the users. According to the framework, the cloud stores the IoT devices' behaviours and furthermore updates them in a convenient way. Then, the FNs are set in the organization in such a way that the IoT gadgets can convey to get administrations in minimal time. The FNs moreover tackles the issues and offer kinds of help to the IoT gadgets in vicinity. The FNs are associated with one another in a wired/remote way. The IoT gadgets at the bottom layer are associated with the FNs in their vicinity by making use of BSS and GW. They sends and gets information remotely by utilizing 4 G/LTE/3 G/WiMAX. The BSs and GT likewise sends and gets information remotely by utilizing 4 G/LTE/3 G/WiMAX. It is outside the purview of this study to deploy fog nodes and cloud nodes wherever necessary. We just pay attention to how the layers are connected and how the various network elements communicate.

### 5.2. Network's Data Classification Setup

**Figure 4:**
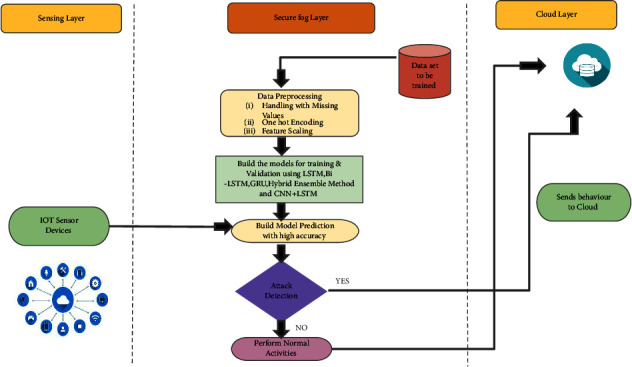
Data preprocessing steps.

On establishing network connections at each tier, the FNs are empowered with AI (utilizing DL model) to anticipate the behaviour of the IoT devices. The DL model is carried out at the computing module CMFN of the FN and the model is chosen based on its highest prediction accuracy. For attack detection, the models are trained in prior with what are considered standard datasets. Before training the model, the datasets undergo with various preprocessing steps for selection of features by means of missing value handling, feature scaling, and one-hot encoding. A diagram depicting the preprocessing procedures is shown in Figure 4, the details of which are explained as follows.

#### 5.2.1. Data Preprocessing

The preprocessing of the dataset is as follows:

1. Handling of missing values: the DL model encounters problems when a sizable fraction of the datasets utilized for classification have missing values. According to the proffered framework, we dealt with the missing values by eliminating the columns or rows which have zeros or null values. Subsequently, we additionally search for mean and median techniques by supplanting the missing values with mean or median. Notwithstanding, it is just employed for numeral data.
2. Feature scaling: datasets having features of variable types and values will need to have their features scaled to meet the specifications. Normalization and standardization are two of the most well-known methods. To put it simply, the normalization method is used if the data does not have a Gaussian distribution, and the standardization method is used otherwise. The term “normalization” refers to the process of adjusting the absolute values of attributes in a dataset to create a consistent scale without affecting the relative variances between values. In the process of “standardization,” the mean is lowered to zero and the standard deviation is raised to one.
3. One-hot encoding: since the DL model cannot process categorical information, it is necessary to transform the dataset's categorical features into numeral data using one-hot encoding in order to improve prediction. The categorical data is transformed using this method into a categorical new vector, which maps to an integer and each integer is represented by a binary vector.
4. Attribute/feature selection: the dataset that comprises attributes of which some attributes affect the arrangement of attacks that should be taken out from the dataset. Comparably, attributes containing zero values can likewise be eliminated to improve attribute choice. Using feature ranking and feature correlation, we remove features which degrades the capability of detection of DL algorithms. Figure 4 shows the information prehandling, preparing, and testing that is performed involving DL model in FN.

#### 5.2.2. Splitting Dataset

Here, the dataset is partitioned into training and test dataset after completion of data preprocessing. The DLM is trained on training dataset and model accuracy of prediction is tested on test set. The partition process of considered dataset is 80 : 20 ratio.

#### 5.2.3. DLM Used for Prognostication of Attack Behaviour

The fog nodes CMFN are trained on training dataset after partition using DLMs. In the following sections, we considered various DLMs for IoT devices behaviour prediction [1]. The models used are DNMLP, LSTM, Bi-LSTM, GRU, CNN + LSTM, and HEM.

*(1) DNMLP*. Fundamentally, an input layer, an output layer, and a hidden layer with an arbitrarily chosen number of hidden layers make up a DNMLP architecture [10]. Except for the input layer, every neuron in this layer uses a nonlinear activation function. Information flows forward in the DNMLP in order to be described, and the neurons are also set up with a backprop algorithm. The DNMLP design's first step takes into account the sum of information values *i*_*k*_ multiplied by *w*_*k*_:

(1) $$\begin{array}{c} i_{k}w_{k}=i_{1}w_{1}+i_{2}w_{2}+\cdots +i_{n}w_{n}\text{.} \end{array}$$

In the subsequent advance, bias *b*_*i*_ is added as follows:

(2) $$\begin{array}{c} Y=i_{k}w_{k}+b\text{.} \end{array}$$

Now *Y* value is advanced through the activation function ReLU or Softmax, generally denoted by $\hat{y}$:

(3) $$\begin{array}{c} \hat{Y}=\text{maximum}\left(0,Y\right)\text{.} \end{array}$$

The above function will return zero if *Y* < 0, and if *Y* ≥ 0, the result is just input. Now that the final step loss ${\left(Y-\hat{Y}\right)}^{2}$ has been calculated, it should be limited if it is higher by modifying *w*_*k*_ and *b*, which should be feasible with an optimizer. As a result, the cost function is calculated as $\sum_{i=1}^{k}={\left(Y-\hat{Y}\right)}^{2}$. We arrive at global minima using the backprop technique in a predetermined amount of cycles, and we can consider this to be the success of preparing DNMLP.

**Figure 5:**
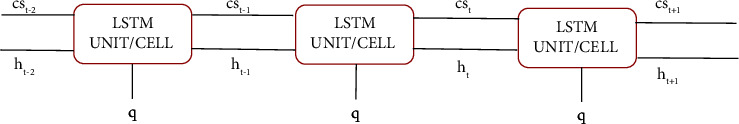
Chain of LSTM units [39].

*(2) LSTM*. LSTM is specifically made to address the issue of RNN's long-term dependencies [38]. It is employed for categorizing data and producing prognostications. Cell state, input gates, forget gates, and output gates make up each LSTM unit. It is employed in language modeling, network anomaly detection, picture captioning, and other processes. Because LSTM can retain data for a long time, it is frequently used to categorize data. A chain of LSTM units can be depicted as in Figure 5.

An LSTM cell's progressive flow is governed by the following equations:

(4) $$\begin{array}{c} e_{t}=sigm\left[\left(w_{e}.a_{t-1}\right)+\left(w_{ep}.p_{t}\right)+b_{e}\right]\text{,} \end{array}$$

(5) $$\begin{array}{c} n_{t}=sigm\left[\left(w_{n}.a_{t-1}\right)+\left(w_{np}.p_{t}\right)+b_{n}\right]\text{,} \end{array}$$

(6) $$\begin{array}{c} x_{t}=\text{Htangent}\left[\left(w_{x}.a_{t-1}\right)+\left(w_{xp}.p_{t}\right)+b_{x}\right]\text{,} \end{array}$$

(7) $$\begin{array}{c} s_{t}=s_{t}^{e}+s_{t}^{n}\text{,} \end{array}$$

(8) $$\begin{array}{c} y_{t}=sigm\left[\left(w_{y}.a_{t-1}\right)+\left(w_{yp}.p_{t}\right)+b_{y}\right]\text{,} \end{array}$$

(9) $$\begin{array}{c} a_{t}=\text{Htangent}\left[\left(s_{t}\right).y_{t}\right]\text{,} \end{array}$$

where *e*_*t*_ is forget gate, *a*_*t*_ is hidden state, *n*_*t*_ is input gate, *x*_*t*_ is cell state, *y*_*t*_ output gate, and *s*_*t*_ is cell vector.

*(3) Bi-LSTM*. It stands for Bidirectional LSTM and works on historical data for extracting spatial features and bidirectional time dependencies [40]. It has been developed for many applications, like protein structure prediction, handwritten recognition, and speech recognition. In the former and future sequences, the best benefits result from the input sequence. In this process, the first layer is given an input sequence, and the next layer is given an input of reverse copy, where the primary and secondary layers are connected with the same layer of output.

*(4) GRU*. GRU is a mechanism of RNN in similar fashion to LSTM but with no output gate [1, 41]. It is considered a variant of LSTM used to overcome the vanishing gradient problem by means of an update and reset gate. Both gates are utilized to regulate the movement of information into and out of memory. GRU outperforms LSTM, which takes longer on large datasets, in comparison. GRU performs better than LSTM for smaller datasets. Speech signal modeling, handwriting recognition, and polyphonic music modeling all make extensive use of GRU. The update gate (*u*) and the reset gate are the two gates that make up the GRU (rs). The calculation of *u* and rs gates at time *t*−1 is illustrated in the following equations:

(10) $$\begin{array}{c} u_{t-1}=\text{sigmoid}\left[\left(W_{u-1}.h_{t-2}\right)+\left(W_{u-1}.p_{t-1}\right)+b_{u}\right], \end{array}$$

(11) $$\begin{array}{c} rs_{t-1}=\text{sigmoid}\left[\left(W_{rs-1}.hs_{t-2}\right)+\left(W_{rs-1}.p_{t-1}\right)+b_{rs}\right], \end{array}$$

(12) $$\begin{array}{c} cs_{t-1}=\tanh\;\left[\left(W_{hs}.p_{t-1}\right)+W_{hs}\left(hs_{t-2}⊙rs_{t-1}\right)+b_{hs}\right], \end{array}$$

(13) $$\begin{array}{c} hs_{t-1}=\left(u⊗cs\right)⊕\left(\left(1-u\right)⊗hs_{t-2}\right)\text{.} \end{array}$$

**Figure 6:**
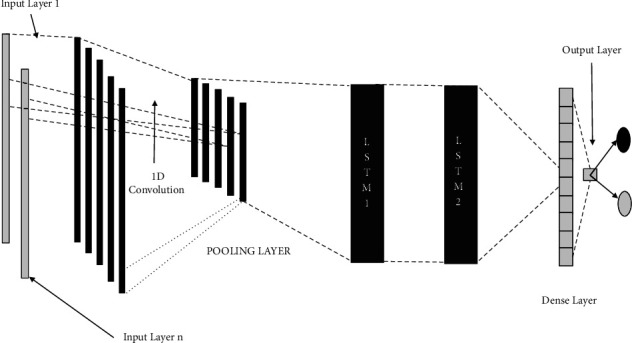
CNN + LSTM architecture.

*(5) CNN* *+* *LSTM*. It is a blended DLM intended for visual time series expectations and text-based classification, such as video depiction and image chaining.  Figure 6 depicts the constructed CNN + LSTM model. The CNN + LSTM engineering consolidates CNN layers for feature extraction from inputs and LSTM layers for time sequence expectation. CNN + LSTM has accomplished upgrades in speech recognition on DNN. It is utilized in visual acknowledgment and elucidation in [42].

**Figure 7:**
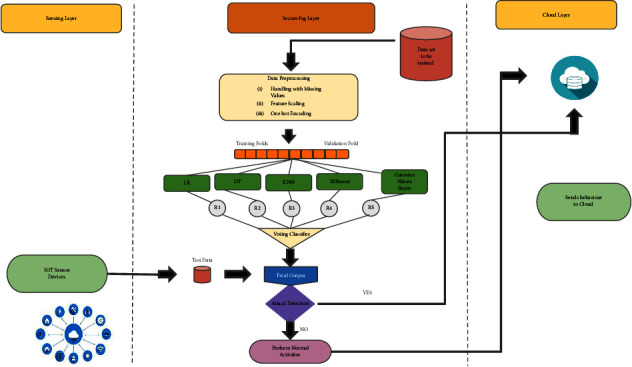
HEM architecture.

*(6) HEM*. In the proposed architecture, a hybrid ensemble method Figure 7 is used for attack detection at FN [25]. This model is constructed into three stages: data preprocessing, hybrid ensemble mechanism, and data gathered from IoT end devices. In the second stage, the hybrid ensemble mechanism is implemented by considering k-fold cross-validation where *k* = 10, which needs to be trained on five different ML algorithms, namely, logistic regression (LR), decision tree (DT), XGBoost, K-Nearest neighbour (KNN), and Gaussian naive bayes (NB). The considered data set is partitioned into *k* parts, of which *k*^*th*^ portion is served as testing set, and the left over *k*−1 part is served for training. On this *k*−1 and *k*^*th*^ part, the above five algorithms are executed collaterally, which obtains five different prediction results denoted as *R*1, *R*2, *R*3, *R*4, and *R*5 that are used for final classing on the voting classifier. In the third stage, the data from IoT end devices is collected at FN as test data, which was tested for classing the attack behaviour.

#### 5.2.4. Dataset Description

The DLMs are assessed on old and novel datasets to distinguish the various attacks and characterize the end client conduct (benign/assailant). The datasets used in this framework for training and testing are discussed as follows:

1. DDoS-SDN: The DDoS-SDN dataset is browsed in Mendeley Data, which includes 104345 records having 23 traits [16]. It is owned to recognize data traffic as harmless or vindictive in light of TCP Syn, UDP flood, and the ICMP attacks. Switch_*i*_*d*, Packet_*c*_ount, byte_*c*_ount, and so on are among the traits. The data-traffic characterization marked 0 for the harmless client and 1 for the malignant client. The 15 customizable properties in the dataset include 14 features and 1 target variable. The binary classification of the target label is 0 (normal/benign user) and 1 (attacker/assaulter). One category trait named protocol, which is one-hot encoded, is present in the dataset.
2. NSL-KDD: The NSL-KDD dataset [17] is constituted into reality, by means of modified and tidied-up variant of the KDD99 from the University of New Brunswick. The NSL-KDD dataset has browsed the Kaggle Repository with 148517 (test and train) records with 43 traits. Out of total records, 77054 records are normal and 71463 are anomalies which clearly exhibits its balanced nature. The dataset is converted into CSV format. Attributes that have no impact on the dataset were discarded and thought about just 19 elements. The categorical traits protocol_type, service, and flag were one-hot encoded to refashion over into mathematical traits. The protocol_type attribute is of three types, namely, icmp, tcp, and udp, the service attribute is of 70 types and flag is 11 different types. After one-hot encoding dataset features were increased to a hundred in number. This dataset contains two class labels, namely, normal and anomaly. There are four unique attack types in the NSL-KDD dataset: denial of service (DoS), user to root (U2R), probe, and remote to local (R2L).
3. UNSW_NB15: The third dataset is UNSW_NB15 is chosen from UNSW_NB15 || Kaggle, developed by Intelligent Security Group, UNSW, and Canberra, Australia store, with 2540044 instances and 49 features [18]. In this work, the dataset considered from Kaggle contains 257673 instances including UNSW_NB15_training-set.csv and UNSW_NB15_testing-set.csv with 45 features including 01 class label. It was customized to 43 features including 01 class label. The categorical feature proto comprised of 133 unique labels and only 15 unique labels are considered in this work and remaining are discarded. So that a total of 242432 instances are taken into account. It describes in total of nine categories as attackers (fuzzers, analysis, backdoors, DoS, exploits, generic, reconnaissance, shellcode, and worms) with 164673 instances and one as normal with 93000 instances. The target label is divided into two categories as 0 (normal) and 1 (attacker).
4. IoTID20: The IoTID20 is the fourth dataset used in the proposed work, with 86 columns, in which three are label features and 625783 instances, which is customized to 68 columns using correlation [19]. The three label features are named as binary, category, and subcategory. The binary label feature is distributed as normal and anomaly with 40073 and 585710 records, respectively. The category label feature is distributed as normal, DoS, mirai, MITM, and scan with 40073, 59391, 415677, 35377, and 75265 records. The last subcategory is distributed as ten label features (normal, DoS, mirai ack flooding, mirai Brute Force, mirai HTTP flooding, mirai UDP flooding, MITM, scan host port and scan port OS) with 40073, 59391, 55124, 121181, 55818, 183554, 35377, 22192, and 53073 records. The main advantages of the IoTID20 dataset; it imitates a cutting edge pattern of IoT network correspondence; it is among the couple of openly accessible IoT intrusion detection dataset.

### 5.3. Deploying Deep Learning Models and Configuring the Network

We select the DL model after completing the training of the above models on the considered datasets, resulting in high accuracy in prognosticating the behaviour of IoT end devices as normal or malicious. The maximum accuracy attained after training and testing each model is used to make the model selection. Now that the chosen model has been deployed, the entire architecture is prepared for real-time processing where the FN and IoT end devices communicate with one another on the CMFN in the fog layer of fog nodes. The finest algorithm for choosing DL models is Algorithm 1. The network configuration and DL model installation at the fog layer is shown in Algorithm 2.

**Theorem 1 .**

*The IoT device iot_i_s* total result time (TRT) is denoted by TRT_ioti_.

**Proof**

Consider an IoT device *iot_i_* nearer to fog node FN_*i*_ which sends REQ to FN_*i*_. The time to send REQ is formulated as follows:

(14) $$\begin{array}{c} T_{iot_{i}-FN_{i}}=T_{iot_{i}-BSS}+T_{BSS-GW}+T_{GW-FN_{i}}\text{,} \end{array}$$

where *T*_*iot*_*i*_−*BSS*_ is the request time from *iot*_*i*_ to BSS, *T*_BSS−GW_ is the request time from BSS to GW, and *T*_GW−FN_*i*__ is the request from GW to FN_*i*_. The execution time to process the request by FN_*i*_ is represented as *T*_execution_*FN*_*i*___ as follows:

(15) $$\begin{array}{c} T_{{\text{execution}}_{FN_{i}}}=T_{{\text{queue}}_{FN_{i}}}+T_{{\text{compute}}_{FN_{i}}}\text{,} \end{array}$$

where *T*_queue_*FN*_*i*___ is the time spent in waiting queue and *T*_compute_*FN*_*i*___ is the computation time for processing the request to obtain the outcome. Then, outcome is passed to the *iot*_*i*_ with a time of *T*_*FN*_*i*_−*iot*_*i*__:

(16) $$\begin{array}{c} T_{FN_{i}-iot_{i}}=T_{FN_{i}-GW}+T_{GW-BSS}+T_{BSS-iot_{i}}\text{,} \end{array}$$

where *T*_FN_*i*_−GW_, *T*_GW−BSS_, and *T*_BSS−*iot*_*i*__ are all the time to send outcome to *iot*_*i*_. Therefore, the TRT is calculated as follows:

(17) $$\begin{array}{c} TRT_{iot_{i}}=T_{iot_{i}-FN_{i}}+T_{{\text{execution}}_{FN_{i}}}+T_{FN_{i}-iot_{i}}\text{.} \end{array}$$

### 5.4. Identification of Assault

From the above two algorithms, it is depicted the way in which a DLM model is chosen and introduced in the FNs. A while later, the IoT devices began correspondence with the FNs in nearness for getting administrations. Notwithstanding, after the communication the proposed model predicts the way of behaving of the IoT devices from recorded CB. As the CMFN_*i*_ is empowered with DLM, it can predict the way of behaving of the IoT devices (malicious or benign). On completion of classification, the refreshed behaviour of the IoT device at FN is transferred to the cloud CN for accumulation and refurbish.  Algorithm 3 shows the classing behaviour of IoT end devices by FN.

**Theorem 2 .**

*Aiot*_*i*_ *IoT device's communication behaviour detection time (CBDT) is expressed asCBDT*_*iot*_*i*__.

**Proof**

Allow *l* number of CB for *l* number of IoT gadgets in the FN queue. Consequently, the CBDT_iot_i__ of an IoT device *iot*_*i*_ of a fog node *FN*_*i*_ is computed as follows:

(18) $$\begin{array}{c} CBDT_{iot_{i}}=T_{{\text{queue}}_{cb_{i}}}+T_{\text{prognostication}}\text{,} \end{array}$$

where *T*_queue_*cb*_*i*___ is the time spent in the FN_*i*_ waiting queue of the IoT device communication behaviour and *T*_prognostication_ is the time needed to detect the IoT device communication behaviour by FN_*i*_. □

The time complexity to test (18) depends on its execution, i.e., the model we selected for deploying on FN_*i*_. It is clearly observed from Section 6.2 that the finest DLM obtained for classifying the behaviour of IoT end devices is LSTM model which is deployed on FN_*i*_. The complexity of LSTM model with multiple LSTM layers always depends on its implementation. Generally, any model with neural networks is tested by means of an onward pass. To obtain the complexity for any LSTM network with layers we need to consider the LSTM units which are connected in a recurrent manner.

Equations (4)–(9) which represent the onward pass of LSTM layer generate its time complexity on *n*_*t*_ as *O*(*n*(*d*+*n*+2)) where *n* and *d* are dimensions. The computation of *e*_*t*_, *x*_*t*_, and *y*_*t*_ are same as *n*_*t*_ and, thus, the complexity will be *O*(4*n*(*d*+*n*+2)). Considering the cell vector *s*_*t*_ and the hidden state *a*_*t*_ time complexity of each is *O*(2*n*). Hence the total time complexity for single LSTM layer onward pass is *O*(4*n*(*d*+*n*+3)). According to the (18) the complexity of CBDT_iot_i__ also depends on *T*_queue_*cb*_*i*___ for the *cb*_*i*_ insertion into the queue and is (1). Hence, the total complexity for CBDT_iot_i__ in a single onward pass is only *O*(4*n*(*d*+*n*+3)).

### 5.5. Behaviour Update at Cloud

The cloud node CN updates the IoT device information table with the updated behaviours after receiving the behaviour of IoT end devices from the FN. The device information table is updated after receiving the responses from FN_*i*_. The behaviour update at cloud node CN is shown by Algorithm 4. Here, the storage operations which are external to the main memory depend on the table structure maintained, the type of indexing that is being supported, the number of disc accesses that are done, the complexity of the query, etc.

### 5.6. Network Update at FN

Here, the cloud CN transmits the smart gadgets TL_cb_*i*__ to the FNs via transmission links GW_CN_, GW_CN_ to BSS, BSS to GW_*i*_, and GW_*i*_ to FN_*i*_ to update the local tables at FN closest to BSS. Further if transmission occurs among neighboring FNs, it is done only when the behaviour is verified using the local database. If it is discovered to be an assaulter, it terminates additional interaction with the network's adjacent nodes. The network refresh at FN is shown by Algorithm 5.

**Theorem 3 .**

*The time to refurbish/update the attacker/assaulter behaviour at FN (TTR) is the total amount of time required by the cloudCNto update IoT end device behaviour at FN.*

**Proof**

Consider at time *T*, *h* attacker devices prognosticated behaviours are denoted as {*TL*_*cb*_1__, *TL*_*cb*_2__,…, *TL*_*cb*_*h*__} for *l* IoT devices. These prognosticated behaviours are sent as message Msg to the FNs. To calculate TTR, to send Msg from CN to FN is represented as:

(19) $$\begin{array}{c} TTR=T_{CN-GW_{CN}}+T_{GW_{CN}-BSS}+T_{BSS-GW_{i}}+T_{GW_{i}-FN_{i}}\text{,} \end{array}$$

where *T*_*CN*−*GW*_*CN*__ is the time required to send the message Msg from *CN* to *GW*_*CN*_, *T*_*GW*_*CN*_−*BSS*_ is the time required to send the message *M* from *GW*_*CN*_ to *BSS*, *T*_*BSS*−*GW*_*i*__ is the time required to send the message Msg from *BSS* to *i*^*th*^ *GW*, and *T*_*GW*_*i*_−*FN*_*i*__ is the time required to send the message Msg from *i*^*th*^ *GW* to *i*^*th*^ *FN*.

## 6. Performance Evaluation

To test how well the proffered framework works, Python 3 is used as a software requirement, and the core i7-11370 CPU, 3.30 GHz clock speed, and 16 GB RAM are used as hardware requirements. The framework is implemented with various DLM models on four datasets, resulting in different accuracies. The accuracy (Accr), precision (P), recall (R), and F1-Score (*F*1_*S*) of DLM models are calculated using confusion matrix parameters, where true positive is (*T*_*P*), true negative is (*T*_*N*), false positive is (*F*_*P*), and false negative is (*F*_*N*):

(1) Accuracy (Accr): Accuracy is characterized by the number of correct predictions obtained from the observed values. The notation is as follows:
  (20) $$\begin{array}{c} \text{Accr}=\frac{T\_P+T\_N}{T\_P+T\_N+F\_P+F\_N}\text{.} \end{array}$$
(2) F1-Score: The harmonic mean of recall and precision is used to reckon the F1_S in order to provide more accurate results. Below is a representation of an F1_S:
  (21) $$\begin{array}{c} F1\_S=\frac{2\times P\times R}{P+R}\text{.} \end{array}$$
(3) Precision: Precision is a model's consistency in categorizing the model as positive and is denoted as follows:
  (22) $$\begin{array}{c} P=\frac{T\_P}{T\_P+F\_P}\text{.} \end{array}$$
(4) Recall: Recall is the ability how well a model can identify positive samples and below is the representation:
  (23) $$\begin{array}{c} R=\frac{T\_P}{T\_P+F\_N}\text{.} \end{array}$$

### 6.1. Attack Simulation Using DLMs

Python 3 DNMLP, LSTM, Bi-LSTM, GRU, CNN + LSTM DL models, and HEM are used to discover the most accurate model. The DDOS-SDN dataset, NSLKDD dataset, UNSW-NB15 dataset, and IoTID20 dataset, [16, 19, 42] are utilized for prognosis. Anaconda's Keras module on TensorFlow is used for deep learning implementation. The SkLearn Package was used to implement and evaluate the hybrid ensemble model. The matplotlib is used obtain graphs on accuracy and loss performance.

**Figure 8:**
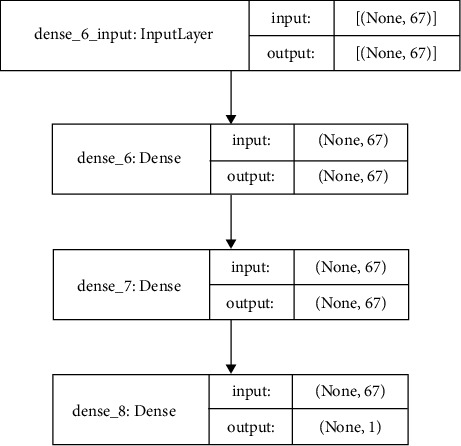
Constructed DNMLP model.

**Figure 9:**
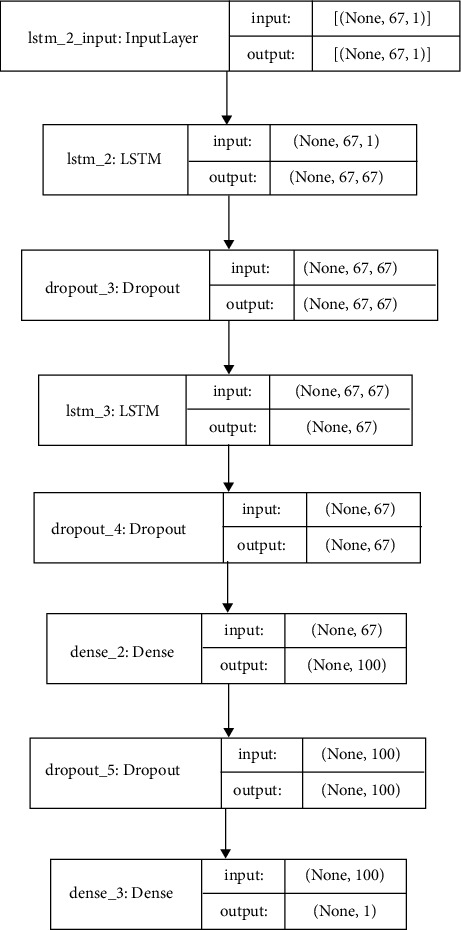
Constructed LSTM model.

**Figure 10:**
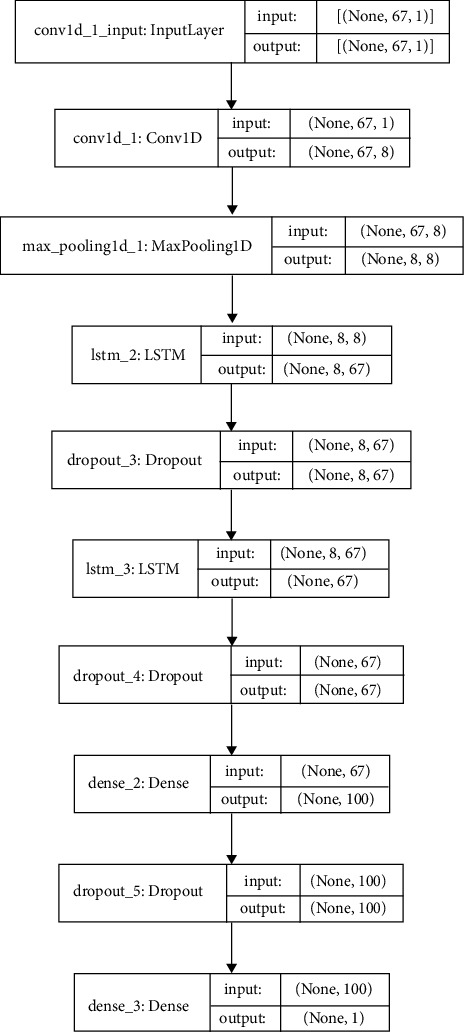
Constructed CNN + LSTM model.

Using the DDOS SDN dataset, NSLKDD dataset, UNSW-NB15 dataset, and IoTID20 dataset, we trained and evaluated the abovementioned models for binary classification (normal or attacker). Different attacks are involved with considered datasets [16, 19, 42] and are utilized to recognize the ability of DNMLP, LSTM, Bi-LSTM, GRU, CNN + LSTM DL, and HEM models for attack identification. In our work to prognosticate the attacks, some features from the considered datasets are discarded on the basis of high correlation among the traits, or those features that don't affect the prognostication. On the expulsion of these attributes, the computation burden is lowered, and thus the framework is built with vital information. Utilizing a standardization strategy, the dataset is scaled on different traits for the fluctuating sizes of values and divided into two proportions of 80 : 20 as train and test data. The point of apportioning the dataset in the proportion of 80 : 20 is to prepare the model with sufficient data and to corroborate the model with suitable data. To procure the most accurate trained model in the proposed framework using the LSTMDL model, we considered a mini batch of 32 with 100 epochs on the Adam optimizer using the learning rate (LR) of 0.001 and considered beta values as arguments for the first- and second-moment exponential decay rate estimates as 0.9 and 0.999, which prevents an adverse effect on optimization for binary classification. The callback function on early stopping is called on TensorFlow, which keeps track of flow to decide the termination condition on validation loss. For the datasets under investigation, NN is built using Keras on TensorFlow using the aforementioned models. In this work, we constructed a model for DNMLP as shown in Figure 8 on new IoTID20 dataset and also a model is built using LSTM is shown in Figure 9, in a similar way the model is built on Bi-LSTM and GRU. On the same dataset, the model is also built on CNN + LSTM as shown in Figure 10. We used ReLU as the activation function in the dense layers of DL models and sigmoid activation function in the output layer as we performed binary classification. Using a stacking approach with a voting classifier, the model is built on HEM using the same IoTID20 dataset as discussed in Section 5.2.3. As described above for constructed models on the IoTID20 dataset, in the same way, constructed models were created on the remaining datasets after performing one-hot encoding. The sequential model is used to create NN with Keras, and it accepts the result of each layer as a contribution to the subsequent layer that uses the add-on model. The dense from the Keras package was used to determine the completely associated layer.

In the implementation of HEM using the stacking approach as discussed in Section 5.2.3, after preprocessing, in stage 1, five algorithms such as LR, DT, XGBoost, KNN, and NB are imported from sklearn machine learning library. In the second stage, we used a voting classifier by importing the package using “sklearn.ensemble import VotingClassifier” for final classing.

### 6.2. Results and Discussion

**Figure 11:**
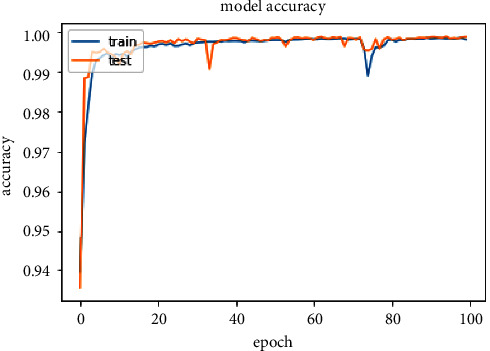
Model accuracy of IoTID20.

**Figure 12:**
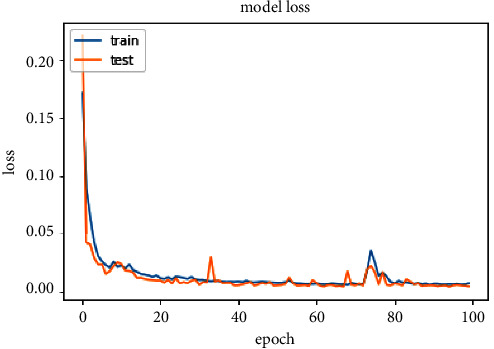
Model loss of IoTID20.

**Figure 13:**
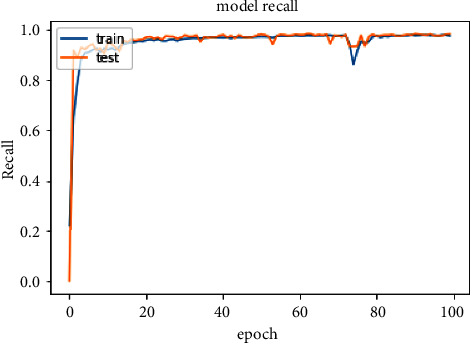
Model recall of IoTID20.

**Figure 14:**
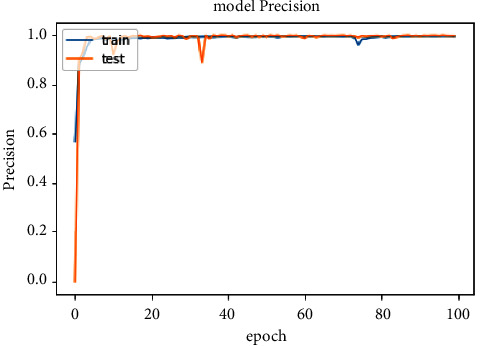
Model precision of IoTID20.

The accuracy of the DL models for binary classing on four datasets is evaluated. The experiment evaluation revealed that a LR of 0.001, a mini-batch of 32, and 100 epochs produced the best performance accuracy. The best performance accuracy over all the datasets is obtained with the LSTMDL model as shown in Table 2 and the model accuracy, model loss, model recall, and model precision graphs of the IoTID20 dataset are shown in Figures 11–14. As IoTID20 is a novel dataset on which only ML models are implemented in previous studies [43], in Section 6 we focused on DLM models on the IoTID20 dataset, whose upshots are depicted in graphs. The execution measures for each model on the datasets taken into consideration for binary classification are shown in Table 2. For the IoTID20 dataset, LSTM achieves better accuracy (99.88%) with precision (99.77%), recall (98.4%), and F1_S (99.08%). The discharge of HEM is comparable to that of LSTM and it outperforms the Bi-LSTM, GRU, CNN + LSTM, and MLP models. With UNSW-NB15 dataset, LSTM achieves better performance (94.11%) with precision (95.87%), recall (94.47%), and F1_S (95.16%). Bi-LSTM performs like LSTM and it outperforms the GRU, CNN + LSTM, HEM, and MLP model. With NSLKDD dataset, LSTM achieves better accuracy (99.12%) with precision (99.22%), recall (99.08%), and F1_S (99.15%). MLP performs like LSTM and it outperforms the Bi-LSTM, GRU, CNN + LSTM, and HEM models. With DDOSSDN dataset, LSTM achieves better accuracy (99.7%) with precision (99.6%), recall (99.64%), and F1_S (99.62%). The discharge of GRU and Bi-LSTM performs like LSTM and they outperform the CNN + LSTM, HEM, and MLP models. For binary classification, excluding DDOSSDN dataset, GRU did not perform well. In implementing GRU, we used dropout mechanism at every stage of constructing model. So, we discarded dropout mechanism and implemented L2 regularization in GRU for better performance. On contrast with all the models on the considered datasets, the false-positive rate (FPR/FAR/1-specificity) on LSTM may not outperformed at its best, but on overall comparison LSTM proved to be performed well with FPR.

**Figure 15:**
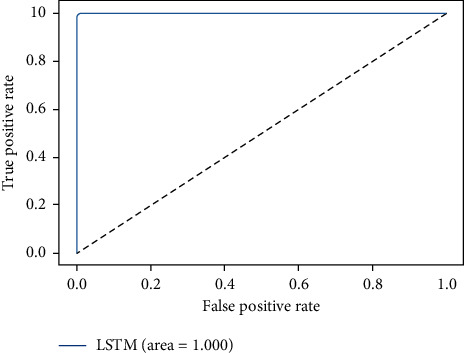
DDOS-SDN_LSTM_ROC-AUC.

**Figure 16:**
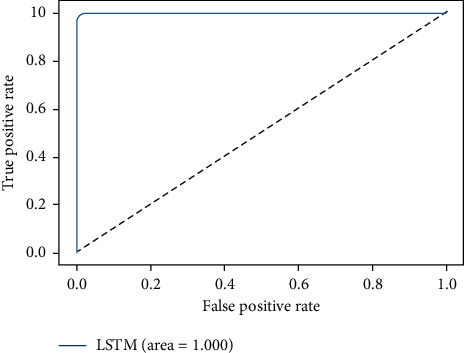
NSL-KDD_LSTM_ROC-AUC.

**Figure 17:**
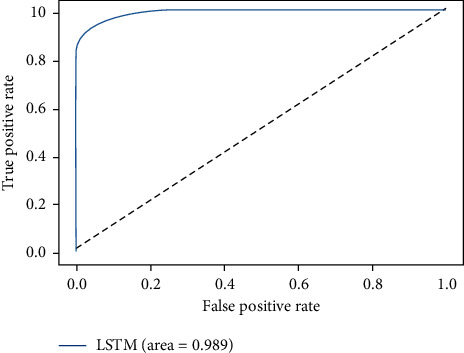
UNSW-NB15_LSTM_ROC-AUC.

**Figure 18:**
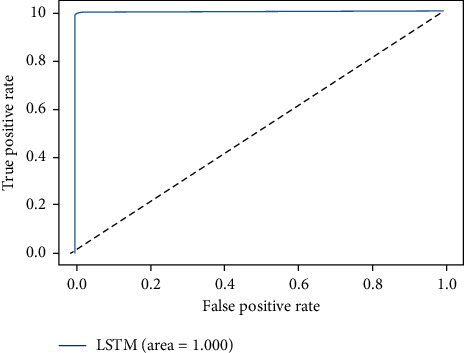
IoTID20_LSTM_ROC-AUC.

The ROC-AUC score of LSTMDLM on all four considered datasets are shown in Figures 15–18. ROC curves are attained by marking out T_P rate (TPR/Recall) versus FPR. AUC summarizes the ROC curve and takes the value between 0 and 1 where one indicates the classifier's exactness in prediction and zero, otherwise. It is evident from above graphs in Figures 15–18 that LSTMDLM exhibited higher AUC score which indicates the ability to classify positives and negatives exactly. Remaining algorithms on all four datasets also showed AUC score between 0.98 and 1.

**Figure 19:**
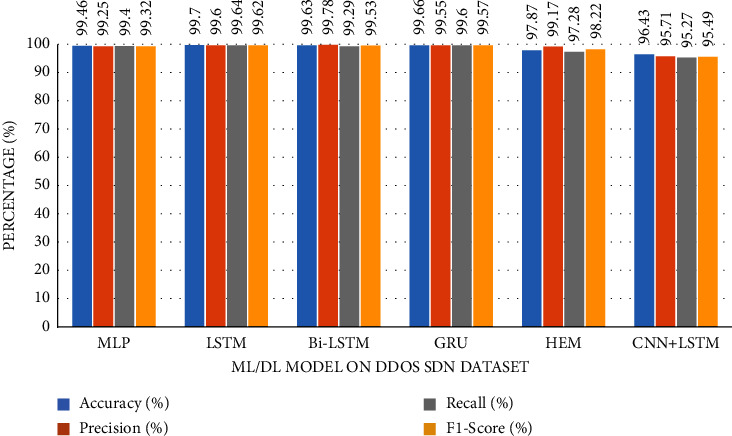
Metrics of DDoS-SDN dataset.

**Figure 20:**
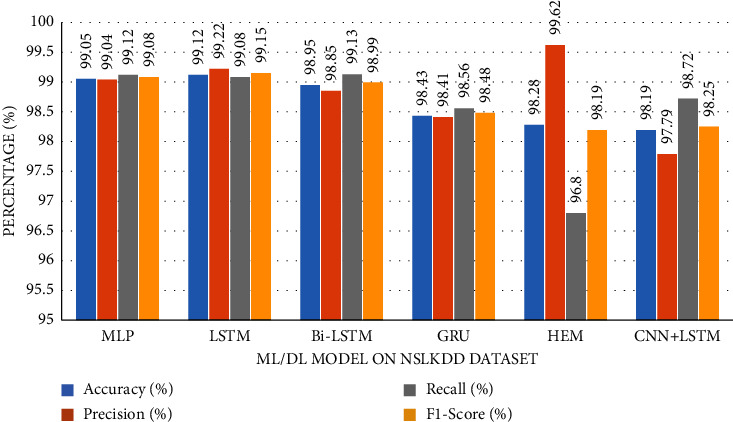
Metrics of NSL-KDD dataset.

**Figure 21:**
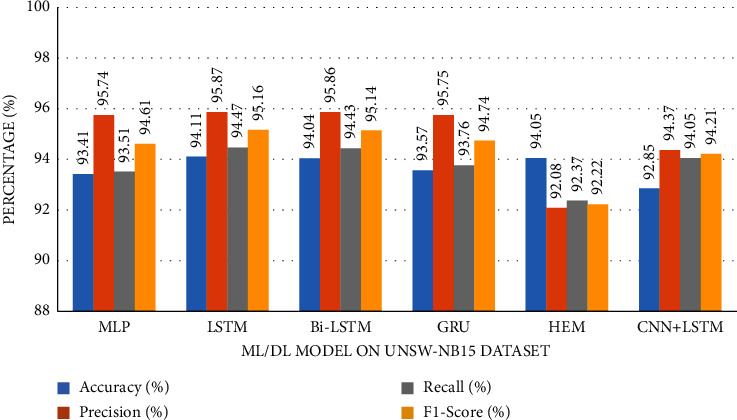
Metrics of UNSW-NB15 dataset.

**Figure 22:**
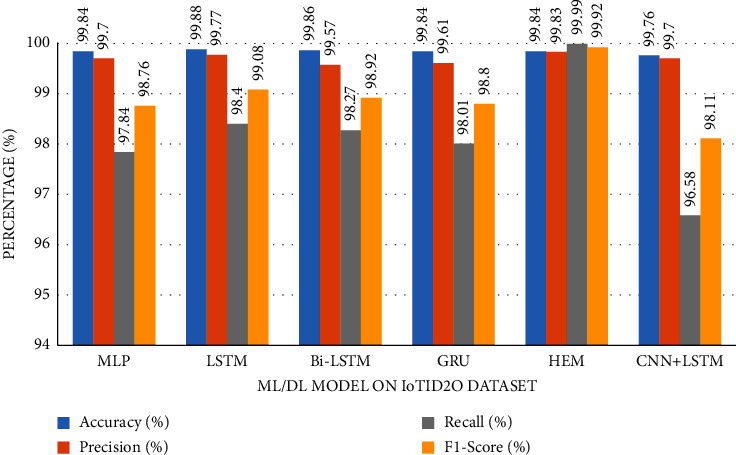
Metrics of IoTID20 dataset.

**Figure 23:**
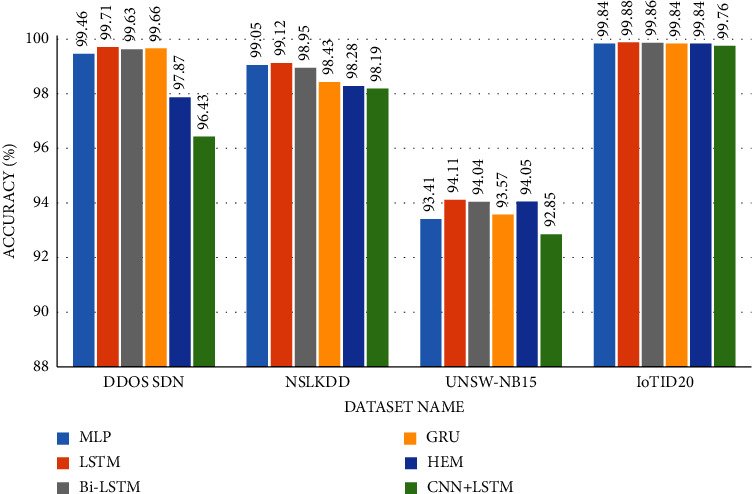
Accuracies of all DLMs on considered datasets.

The study of execution measures amid the DLMs and HEM on the considered datasets is depicted in Figures 19–22. In terms of accuracy, LSTM performed better than the considered DLMs and HEM, as shown in Table 2 with bold values. The accuracies of all DLMs and HEM with binary classing are shown in Figure 23. Hence the LSTM model is prognosticated to be greater in rank compared to all others.

Utilizing datasets, we trained and evaluated DLMs and HEM model for binary classification and found LSTMDLM showed preferred exactness over any remaining models in anticipating the way of behaving of the IoT end devices as normal or attack. On using balanced dataset, accuracy is only considered to be an essential measure in assessing a model. But in this work, excluding NSLKDD, the other remaining datasets are imbalanced, hence there is possibility of having more *F*_*P* and *F*_*N*. In these circumstances, it is smarter to pay attention to the other execution measures like precision, recall, and *F*1_*S*. Recall, in all actuality, does just think about *F*_*N* and *T*_*P* and subsequently, recall might be high. Precision really does just consider *F*_*P* and *T*_*P*, it might endure with low worth. The *F*1_*S*, will have its significance to choose the presentation of the model furthermore. It is obviously clear by the outcomes showing the most elevated worth of *F*1_*S* (99.62%, 99.15%, 95.16%, and 99.08%) on DDoS-SDN (Mendeley Dataset), NSLKDD, UNSW-NB15, and IoTID20 datasets with LSTMDLM as shown in Table 2.

The simulation condition is configured as a three level framework with one cloud server coupled to numerous fog nodes to investigate the expandability problem. We make the assumption that the closest fog nodes are connected to 10–100 IoT devices (smart gadgets) in the final layer. For instance, if there is 1 fog node and 10 smart gadgets, then 10 smart gadgets can connect directly to the fog node. If there is more than 1 fog node, then the number of fog nodes splits the number of smart gadgets equally to provide the required service. Therefore, one fog node will provide aid to 5 smart gadgets if there are 2 fog nodes. In this case, we'll assume that a smart gadget links to the fog node and produces one sample (row). The fog node then processes this sample to forecast behaviour (attack/assault or normal/benign). The average CBDT from the aforementioned examination using DNMLP is discovered to be 0.0000672 seconds for 10 smart gadgets, 0.0024924 seconds for 10 smart gadgets for LSTM, 0.004164 seconds for 10 smart gadgets for Bi-LSTM, 0.0021 seconds for 10 smart gadgets for GRU, 0.000476 seconds for 10 smart gadgets for CNN + LSTM, and 0.008 seconds for HEM, respectively. In this experiment, we looked at how the number of smart gadgets compared to the number of fog nodes might affect the time it takes to identify behaviour. Behaviour detection time (BDT) is the period of time during which fog nodes using DNMLP, LSTM, Bi-LSTM, GRU, CNN + LSTM, and HEM can determine whether a certain number of smart gadgets are benign or an assaulter. The variables and values for the NW simulation are displayed in Table 3.

**Figure 24:**
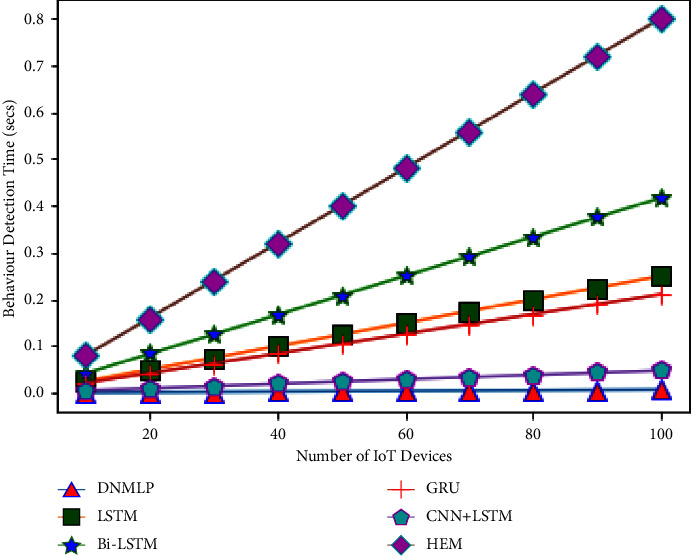
Examination of CBDT for different models having 1 fog node in the NW.

**Figure 25:**
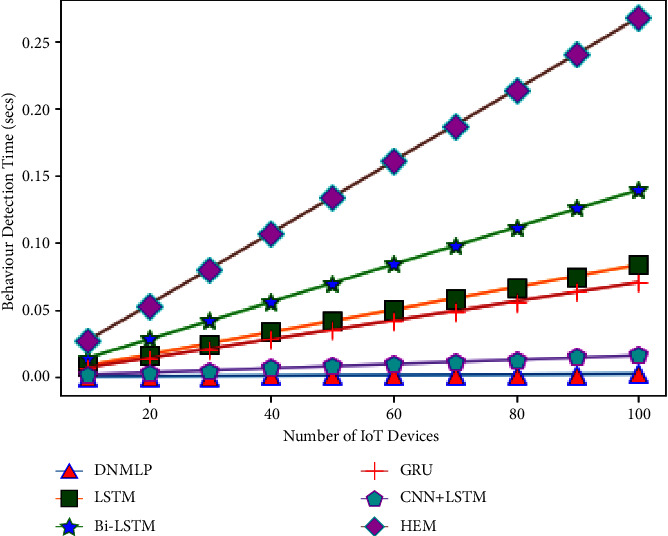
Examination of CBDT for different models having 3 fog nodes in the NW.

**Figure 26:**
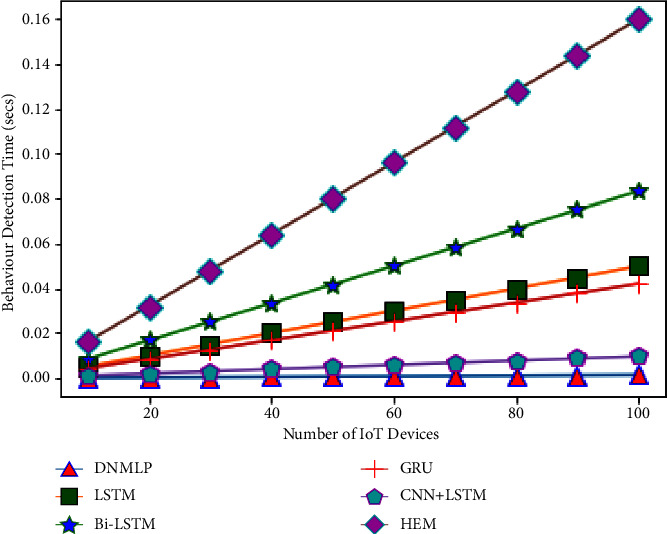
Examination of CBDT for different models having 5 fog nodes in the NW.

**Figure 27:**
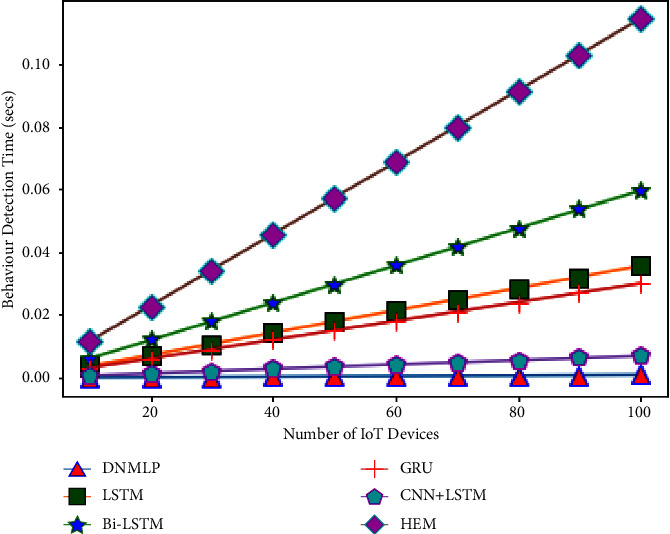
Examination of CBDT for different models having 7 fog nodes in the NW.

**Figure 28:**
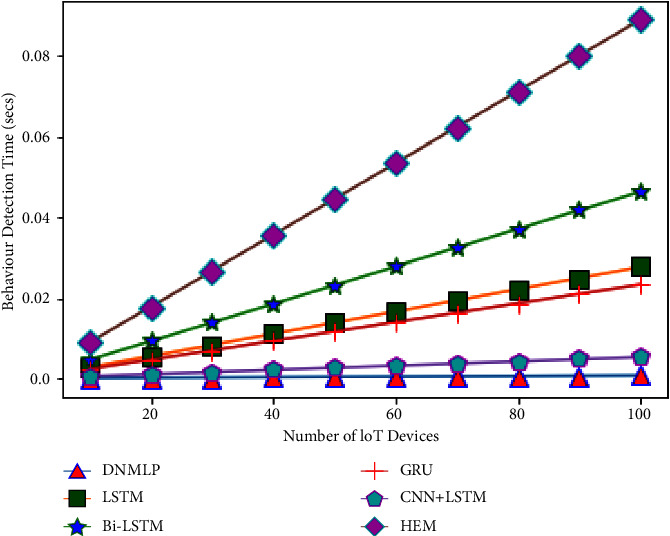
Examination of CBDT for different models having 9 fog nodes in the NW.

From Figures 24–28, it is apparent that as the number of Internet of Things grows in the NW, so does the time it takes for all IoT devices to detect their behaviour. The result is depicted in Figure 24 when there are 1 fog node in the NW and 10–100 IoT devices. According to this graph, DNMLP has a faster time to detect behaviour than LSTM, Bi-LSTM, GRU, CNN + LSTM, and HEM. The DNMLP, LSTM, Bi-LSTM, GRU, CNN + LSTM, and HEM average CBDT for 100 IoT devices were determined to be 0.003695 sec, 0.22902 sec, 0.1155 sec, 0.02618 sec, and 0.44 sec, respectively. The result is shown in Figure 25 when there are 3 fog nodes in the NW and 100 smart gadgets overall. According to this graph, DNMLP has a faster time to detect CB than LSTM, Bi-LSTM, GRU, CNN + LSTM, and HEM. The average CBDT for 100 IoT devices is determined to be 0.001232 seconds for DNMLP, 0.045694 seconds for LSTM, 0.0385 seconds for CNN + LSTM, and 0.146666 seconds for HEM. The outcome is shown in Figure 26 when there are 5 fog nodes in the NW and 100 smart gadgets in total. According to this graph, DNMLP has a faster time to detect CB than LSTM, Bi-LSTM, GRU, CNN + LSTM, and HEM. The average CBDT of 100 IoT devices is determined to be 0.000739 sec, 0.027416 sec, 0.045804 sec, 0.0231 sec, 0.005236 sec, and 0.088 sec for DNMLP, LSTM, Bi-LSTM, GRU, CNN + LSTM, and HEM, respectively. The result is shown in Figure 27 when there are 7 fog nodes in the NW and 100 smart devices in total. According to this graph, DNMLP has a faster time to detect behaviour than LSTM, Bi-LSTM, GRU, CNN + LSTM, and HEM. The average CBDT of 100 smart gadgets is determined to be 0.000527 sec, 0.019583 sec, 0.032717 sec, 0.016499 sec, 0.00374 sec, and 0.062857 sec for DNMLP, LSTM, Bi-LSTM, GRU, CNN + LSTM, and HEM, respectively. The result is shown in Figure 28 when there are 9 fog nodes in the NW and 100 smart devices total. According to this graph, DNMLP has a faster time to detect CB than LSTM, Bi-LSTM, GRU, CNN + LSTM, and HEM. The average CBDT of 100 smart gadgets is determined to be 0.00041 sec, 0.015231 sec, 0.025446 sec, 0.012833 sec, 0.002908 sec, and 0.048888 sec for DNMLP, LSTM, Bi-LSTM, GRU, CNN + LSTM, and HEM, respectively.

From the aforementioned findings, it is also shown that communication behaviour detection time is reduced as the number of fog nodes in the network increases.

## 7. Conclusion

This study proposes a DL model-based assault prognostication system for fog-based Internet of Things environment. The network consists of a smart sensing tier, a secure fog tier, and a cloud tier. Following this, a variety of deep learning (DL) models, including DNMLP, LSTM, Bi-LSTM, GRU, CNN + LSTM, and HEM, are assessed to prognosticate the most accurate model with high exactness for installation at the fog nodes. The DDOS SDN, NSL-KDD, UNSW-NB15, and IoTID20 datasets indicate 99.70%, 99.12%, 94.11%, and 99.88% accuracy using the LSTMDL model, respectively. As a result, the fog tier in the NW is installed using LSTM, with every fog node being equipped with the LSTMDL model. The deployed model performs binary classing as two classes, 1 and 0, as the assailant or benign separately and sends the device CB to the cloud for refurbishing. The cloud then sends the misbehaviour data to the fog nodes, each of which is aware of the local attack situation in the fog layer. The individual fog nodes decide whether to communicate with these attacking devices in the future by evaluating their current behaviour. In a fog-based Internet of Things condition, the proffered model will be a finer stratagem against the attacks from securing the fog layer, which conquers the stratagem of deploying the DLMs in the sensing layer. The results of the proposed framework prove that the considered DLMs can be acquired for cybersecurity to identify cyberattacks that prevailed in distinct datasets. Additionally, network simulation is used to demonstrate how well various DL models portray the CBDT in the fog layer. The LSTMDL model outperforms DNMLP in terms of accurately forecasting the attacks, although it takes longer to identify the activity (CBDT) than other models, according to this study. Additionally, it has been discovered that the CBDT decreases as the fog nodes in the NW grows. We will implement a similar strategy for attack forecasting in the future using multiclass classing. Similar to how specific attacks can be discovered by using more recent datasets, new multilayer deep neural network models such as AlexNet, ResNet, VGGNet, DenseNet, and Shufflenet, can be created by prepping the fog nodes with a larger dataset.

## Figures and Tables

**Algorithm 1:**
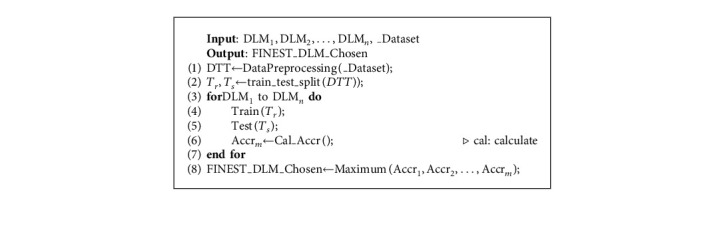
Method of choosing the finest DL model for the fog tier.

**Algorithm 2:**
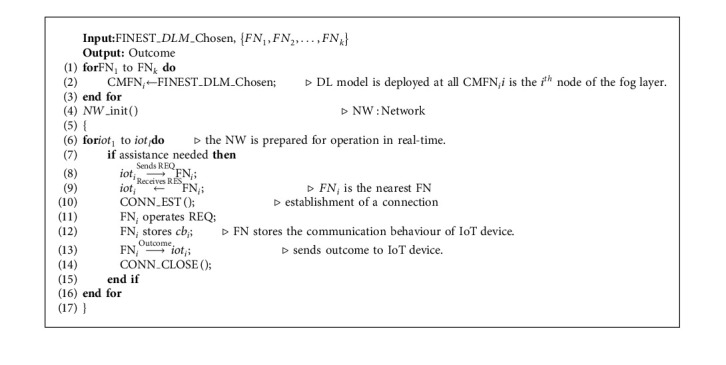
Algorithm for DLM installation and network setup.

**Algorithm 3:**
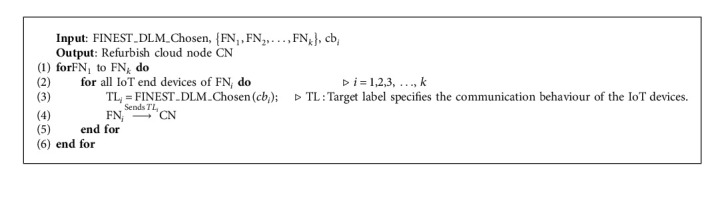
Classing the behaviour of IoT end devices by FN.

**Algorithm 4:**
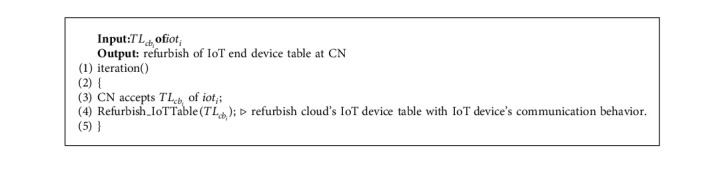
Behaviour update at cloud.

**Algorithm 5:**
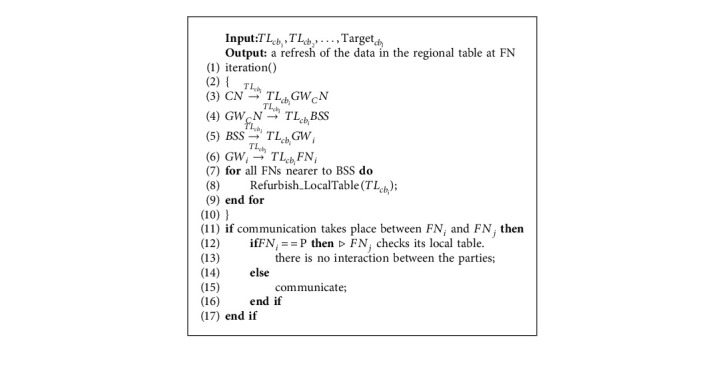
Network update at FN.

**Table 1:** Notes and elaborations.

SI. no.	Notation	Description
i	CN	Cloud server
ii	GW	Network gateway
iii	BS	Base-station
iv	FN	Set of fog nodes
v	FN_*i*_	*i* ^*th*^ fog node
vi	CMFN	Compute module of fog node
vii	MMFN	Fog node's memory module
viii	*iot*_*i*_	*i* ^*th*^ IoT device
ix	*P*	Perpetrator/attacker
x	*I*	Established group of IoT devices
xi	CB	Group of behavior instances
xii	cb	Behavior instance
xiii	*T*	Time instance
xiv	Accr	Accuracy
xv	DTT	Dataset for training and testing
xvi	*T*_*r*_	Train data
xvii	*T*_*s*_	Test data
xviii	TAT	Time of total service
xix	TL	Final Behavior's label
xx	CBDT	Communication behavior detection time
xxi	TTR	Time to refurbish/update FN
xxii	Accr	Accuracy

**Table 2:** Performance metrics of considered DLMs.

Dataset name	ML/DL model	Accr (%)	Precision (%)	Recall (%)	F1_S (%)
*DDOS SDN*	MLP	99.46	99.25	99.4	99.32
	LSTM	**99.7**	99.6	99.64	99.62
	Bi-LSTM	99.63	99.78	99.29	99.53
	GRU	99.66	99.55	99.6	99.57
	HEM	97.87	99.17	97.28	98.22
	CNN + LSTM	96.43	95.71	95.27	95.49

*NSLKDD*	MLP	99.05	99.04	99.12	99.08
	LSTM	**99.12**	99.22	99.08	99.15
	Bi-LSTM	98.95	98.85	99.13	98.99
	GRU	98.43	98.41	98.56	98.48
	HEM	98.28	99.62	96.8	98.19
	CNN + LSTM	98.19	97.79	98.72	98.25

*UNSW-NB15*	MLP	93.41	95.74	93.51	94.61
	LSTM	**94.11**	95.87	94.47	95.16
	Bi-LSTM	94.04	95.86	94.43	95.14
	GRU	93.57	95.75	93.76	94.74
	HEM	94.05	92.08	92.37	92.22
	CNN + LSTM	92.85	94.37	94.05	94.21

*IoTID20*	MLP	99.84	99.7	97.84	98.76
	LSTM	**99.88**	99.77	98.4	99.08
	Bi-LSTM	99.86	99.57	98.27	98.92
	GRU	99.84	99.61	98.01	98.8
	HEM	99.84	99.83	99.99	99.92
	CNN + LSTM	99.76	99.7	96.58	98.11

**Table 3:** Simulation of IoT device communication behaviour at the fog layer.

Sl. no.	Variable	Value
i	Cloud server	1
ii	Fognode count	1–10
iii	Connected device count	10–100
iv	Dataset used	IoTID20
v	Average CBDT of DNMLP for 10 specimens	0.0000672 sec
vi	Average CBDT of LSTM for 10 specimens	0.0024924 sec
vii	Average CBDT of Bi-LSTM for 10 specimens	0.004164 sec
viii	Average CBDT of GRU for 10 specimens	0.0021 sec
ix	Average CBDT of CNN + LSTM for 10 specimens	0.000476 sec
x	Average CBDT of HEM for 10 specimens	0.008 sec
xi	Count of simulations run	10

## Data Availability

Data are available upon request.
